# *In vivo* dose measurement using TLDs and MOSFET dosimeters for cardiac radiosurgery

**DOI:** 10.1120/jacmp.v13i3.3745

**Published:** 2012-05-10

**Authors:** Edward A. Gardner, Thilaka S. Sumanaweera, Oliver Blanck, Alyson K. Iwamura, James P. Steel, Sonja Dieterich, Patrick Maguire

**Affiliations:** ^1^ CyberHeart Inc. Portola Valley CA; ^2^ Voyage Medical Inc. Redwood City CA; ^3^ Department of Radiation Oncology Lübeck Campus of the University Clinic Schleswig‐Holstein Germany; ^4^ Sutter Institute for Medical Research Sacramento CA; ^5^ Department of Radiation Oncology – Radiation Physics Stanford University Palo Alto CA USA

**Keywords:** *in vivo* dosimetry, radiosurgery, arrhythmia, ablation, CyberKnife

## Abstract

*In vivo* measurements were made of the dose delivered to animal models in an effort to develop a method for treating cardiac arrhythmia using radiation. This treatment would replace RF energy (currently used to create cardiac scar) with ionizing radiation. In the current study, the pulmonary vein ostia of animal models were irradiated with 6 MV X‐rays in order to produce a scar that would block aberrant signals characteristic of atrial fibrillation. The CyberKnife radiosurgery system was used to deliver planned treatments of 20–35 Gy in a single fraction to four animals. The Synchrony system was used to track respiratory motion of the heart, while the contractile motion of the heart was untracked. The dose was measured on the epicardial surface near the right pulmonary vein and on the esophagus using surgically implanted TLD dosimeters, or in the coronary sinus using a MOSFET dosimeter placed using a catheter. The doses measured on the epicardium with TLDs averaged 5% less than predicted for those locations, while doses measured in the coronary sinus with the MOSFET sensor nearest the target averaged 6% less than the predicted dose. The measurements on the esophagus averaged 25% less than predicted. These results provide an indication of the accuracy with which the treatment planning methods accounted for the motion of the target, with its respiratory and cardiac components. This is the first report on the accuracy of CyberKnife dose delivery to cardiac targets.

PACS numbers: 87.53.Ly, 87.53.Bn

## I. INTRODUCTION

Atrial fibrillation (AF) is the most common arrhythmia seen in medical practice. It has an estimated prevalence of 1%–2% in the general population and its prevalence increases with age. It is a significant contributor to cardiac morbidity and mortality. Atrial fibrillation confers a five‐fold risk of stroke, with one in five strokes being attributed to the arrhythmia. AF‐related strokes are twice as deadly as non‐AF strokes.^(^ ^1^ ^,^ ^2^ ^)^

Traditionally, arrhythmia is treated by applying radiofrequency (RF) energy to specific locations in the heart to create a scar that blocks aberrant electrical signals. A less common arrhythmia, atrial flutter, is treated by applying the RF to the cavotricuspid isthmus of the right atrium. Paroxysmal AF is treated with a more challenging procedure by applying the RF to the junction of the pulmonary veins and left atrium. The cardiac RF ablation procedure for AF carries with it a significant risk of major complications (4.5%),^(^ ^3^ ^)^ and long‐term efficacy following catheter ablation of AF is unknown. The patient population receiving RF ablation is predominantly younger and healthier than patients with AF at large.^(^ ^4^ ^)^

A small company, CyberHeart (Portola Valley, CA), was established to investigate the use of radiosurgery as an alternative to RF ablation in the treatment of heart rhythm disorders. Sharma et al.^(^ ^5^ ^)^ used the CyberKnife platform (Accuray Inc., Sunnyvale, CA) to create scar tissue in the cavotricuspid isthmus and pulmonary vein atria in normal animal models. The working hypothesis is that radiation, like RF energy, can create localized and discrete scar tissue to block the aberrant electrical signals that cause arrhythmia. The heart is not usually the target of radiation treatment, but rather a critical structure that is avoided during radiation therapy. A clear link has been established between radiation and various cardiac pathologies.^(^ ^6^ ^)^ The evidence is based on whole body (animal studies, atomic bomb survivors) and whole heart (e.g., thoracic CT, Hodgkin's lymphoma treatment) irradiation. However, the CyberKnife is capable of delivering high and precisely focused doses which can spare coronary arteries and the apex of the heart. A radiosurgery approach may be a viable alternative for older patients where the risks of the more invasive RF ablation procedure are greater and the risks of radiation related complications are lower. The CyberKnife has been used on several occasions to treat tumors in or near the heart using stereotactic body radiotherapy (SBRT).^(^ ^7^ ^)^

The heart is a challenging target for radiosurgical ablation for several reasons: the heart structures are difficult to visualize in CT images without contrast agent; the heart is deforming and moving during the treatment due to contractility and respiration; and critical structures are nearby.

In the human heart, the left atrium is immediately anterior to the esophagus and the pulmonary veins straddle the esophagus. Delivering a dose that can create scar in the pulmonary vein ostia without causing complications in the esophagus requires maintaining high spatial dose gradients even during respiratory motion tracking. The amount of heart motion varies with location in the heart, with the apex moving the most and the posterior structures moving the least. The heart motion has two components: deformation from contraction/relaxation of the heartbeat, and respiratory motion of the heart. To date, no commercial system is available for tracking the contractile motion of the heart for the delivery of radiation.

Systems have been developed to track respiratory motion^(^ ^8^ ^–^ ^10^ ^)^ based on monitoring the surface of the chest. All tracking systems track artificial (e.g., fiducials) or natural (e.g., lung tumor silhouettes) surrogates. Any untracked motion of the target relative to the surrogates will degrade the dose delivery accuracy, particularly for plans with high spatial dose gradients.^(^ ^11^ ^)^

**Figure 1:**
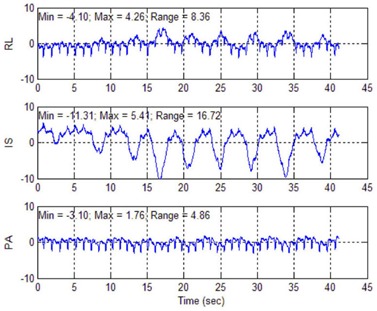
Measurement made using a CARTO system of the motion (in millimeters) of a human left inferior pulmonary vein ostium. The right–left motion is shown in the top panel, the inferior–superior motion is shown in the middle panel and the posterior–anterior motion is shown in the lower panel. Two periodicities were present: that resulting from respiration (period ~ 4 s) and that resulting from cardiac contraction (period < 1 s). The largest component of motion was produced by respiration in the inferior–superior direction.

Measuring the dose during radiosurgical cardiac ablation was considered valuable due to the substantial differences between this treatment and the normal use of radiosurgery. The heart moves in a much more complicated manner than other radiosurgical targets. Motion measurements were made in a human heart using a CARTO tracking catheter (Biosense Webster, Diamond Bar, CA) during a RF ablation procedure. As shown in Fig. 1, cardiac structures move in response to breathing as well as cardiac motion in a complicated pattern. At the same time, high dose gradients were required due to the proximity of critical structures. Traditional *in vivo* dosimetry methods with entrance and exit dose measurements could not be used because of the noncoplanar beam configuration and large number of beam directions of the CyberKnife.

The implantable MOSFET DVS dosimeter^(^ ^12^ ^)^ (Dose Verification System, Sicel Technologies, Morrisville, NC) can be placed at or near the target organ and used to record doses throughout the radiation treatment.^(^ ^13^ ^)^ A variant of the DVS dosimeter has been created (DVS‐HFT) for use in hypofractionation. Scalchi et al.^(^ ^14^ ^)^ have tested this system with phantoms and shown that the measurements agreed with calculations to within 4%. The DVS‐HFT was not used in this study because of its size relative to the canine heart and because of limited availability at the time of the study.

Two types of dosimeters were used to measure the dose to the heart. TLD crystals were implanted on the surface of the heart and MOSFET detectors were threaded into the heart via a catheter. To our knowledge, this is the first report of an *in vivo* measurement of dosimetry at a cardiac target.

## II. MATERIALS AND METHODS

### A. Dosimeter assembly and placement

Two methods of dosimetry were used. Each system presented unique challenges. TLDs were surgically placed in the canine model on the beating heart during a mini‐thoracotomy under general anesthesia. MOSFET sensors were delivered via catheter in both canine and porcine models just prior to ablation and placed as close to the target volume as feasibly possible. The catheter was placed in the coronary sinus which was posterior to the superior aspect of the atrium and inferior to the pulmonary vein targets.

TLD dosimetry was conducted in a single canine model. The TLDs (TLD 100 crystals LiF:MgTi, 3.2× 3.2 × 1 mm, Landauer, Glenwood, IL) were placed in biocompatible, heat shrink tubing (Palladium “Pebax”; Cobalt Polymers, Cloverdale, CA) and 3.2 mm diameter Teflon plugs were placed at each end. An oven was used at 150°C for 3 minutes to shrink the tubing around the Teflon and the TLDs. Twenty‐seven TLDs were used to create four TLD capsules. The TLD capsules were sterilized using a hydrogen peroxide plasma system (STERRAD, Advanced Sterilization Products, Irvine, CA).

**Figure 2:**
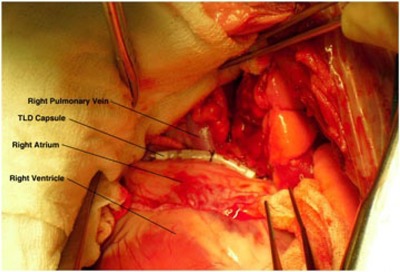
TLD capsule sutured to the epicardial heart surface of a dog. The capsule is located at the junction of the left atrium and right superior pulmonary vein. Caudad to cephalad is the left to right orientation in the picture. The pulmonary vein is the tubular structure emerging from behind the TLD capsule oriented toward the top of the picture. The heart appears at the bottom center of the image, and the boundary between the right atrium (above) and the right ventricle (below) appears as a distinct line above a lighter colored area on the right ventricle.

**Figure 3:**
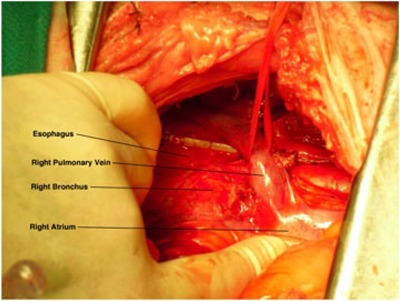
A TLD capsule attached to the anterior midthoracic esophagus of a dog. Cephald to caudad is the left to right orientation.

Two of the TLD capsules were surgically implanted in the canine model prior to CyberKnife treatment, while the others were reserved for calibration. One capsule was sutured to the right posterior epicardial surface at the pulmonary vein left atrial junction (see Fig. 2). Closer placement of the TLD capsule to the target area was limited due to the small right thoracotamy. A second capsule was placed on the esophagus (see Fig. 3). Gold fiducial beads (2 mm diameter, CIVCO, Kalona, IA.) were attached to the epicardium near the pulmonary vein ostia to guide the CyberKnife treatment. Less invasive methods for fiducial placement, such as using the spine for rotational alignment and a temporary fiducial in the heart for tracking, were considered as alternatives. However, alignment uncertainties produced by anatomical differences between the animal and human spine mandated the adoption of the surgical approach for this study.

Forty‐five days after fiducial/dosimeter placement, cardiac‐gated CT scans were acquired. Radiosurgical cardiac ablation was carried out 11 days after CT scanning. It is important to note that the TLD dosimeters were present and visible in the CT scans.

**Figure 4:**
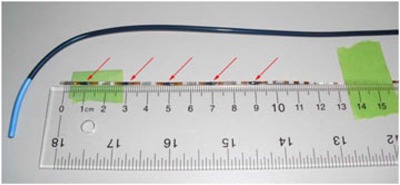
The MOSFET dosimeter array with additional fiducials added midway between the MOSFET sensors and the introducer sheath used to place it in the heart. The MOSFET sensors are dark spots (red arrows), while the platinum‐iridium fiducials appear as bright bands along the array.

Dosimetry using MOSFET sensors was conducted in two canine models and one porcine model. A MOSFET array dosimeter (Linear 5ive Array from Best Medical Canada, Ottawa, Ontario) was used to measure the dose in the coronary sinus. The array consisted of five MOSFET radiation dosimeters attached to a flexible printed circuit (flex) strand at 2 cm intervals with a single fiducial at its tip. To better localize the positions of the dosimeters in X‐ray images, five additional platinum‐iridium fiducials were cemented to the flex midway between the sensors and 1 cm past the proximal sensor (see Fig. 4). The array was selected over a single channel MOSFET (microMOSFET) dosimeter because the length of the array flex (46 cm) was sufficient for catheter placement in the heart from a jugular incision in our canine models. The length of the single channel device (37.5 cm) was not.

A cardiac introducer sheath (6 Fr Flexor Check‐Flo Introducer with Ansel modification, Cook Inc., Bloomington, IN) was modified for placement of the MOSFET array in the coronary sinus. The sheath was cut to a length of 46 cm to allow the MOSFET array to extend its full length, and the tip was heat‐shaped to easily find the coronary sinus ostium in the canine or porcine heart. The distal lumen was plugged with Loctite 5056 biocompatible, UV‐curable silicone to prevent blood from contacting the dosimeter.

Gold fiducial beads (2 mm diameter) were surgically attached to the epicardium near the pulmonary vein ostia to guide the CyberKnife treatment as before. Approximately 40 days after fiducial implantation, CT scans were acquired. The MOSFET dosimeters had not been placed in the animal when the CTs were acquired. Approximately 14 days after CT scanning, the treatments were delivered.

The MOSFET dosimeters were inserted into the coronary sinus of the animals just prior to treatment delivery. A cut‐down was performed at the right jugular vein, and a 12 French CSG introducer sheath was placed. Using fluoroscopic imaging, a 9 French CSG coronary sinus sheath (SafeSheath, Pressure Products, San Pedro, CA) was advanced through the introducer sheath and contrast was injected to find the ostium of the coronary sinus. Once the ostium was found, the coronary sinus sheath was advanced into the coronary sinus. The 6 Fr Flexor catheter containing the MOSFET sensor was then advanced and seated in the lumen of the coronary sinus sheath.

### B. Planning, treatment, and dose calculation

Cardiac‐gated, contrast‐enhanced CT scans were acquired on a GE Lightspeed 16 slice CT scanner, using retrospective reconstruction into 10 cardiac phases (Snapshot Burst+ mode). Cardiac gating was required in order to clearly visualize the left atrium. Iodine contrast (Isovue‐300, Bracco Diagnostics, Princeton NJ) was used to enhance the density of the blood pool. The contrast bolus was timed to preferentially enhance the left heart structures (left atrium and pulmonary veins).

The contrast‐enhanced CT was used to create a CyberKnife treatment plan using the MultiPlan 2.1 software, targeting the left atrium in order to isolate the pulmonary veins. To prevent dose miscalculation due to the presence of contrast agent in the planning CT, the model used for photon/tissue interaction was modified as follows. All tissues with Hounsfield numbers greater than 1000 were modeled as Hounsfield 1000. By clipping the tissue model, the effect of the contrast was eliminated at the cost of underestimating the dose to bony structures. Because the bones (ribs) in the path of the radiation beams were relatively far from the target, little effect was expected. This method was chosen instead of registering the contrast CT with a noncontrast CT to avoid registration errors that would be difficult to avoid due to the cardiac‐gating used in CT acquisition.

The Synchrony respiratory tracking system (Accuray Inc., Sunnyvale CA) was used to create a model for the location of the heart as a function of respiratory phase. As shown in Fig. 1, the heart motion consisted of both respiratory motion and contractile motion of the heart. The chest light sensors of the Synchrony system were insensitive to the contractile motion, so this motion appeared as random error between the model and the heart location. Because 15 points were used to create each Synchrony model, the models approximated the position of the heart at the average point in the cardiac cycle.

Table 1 shows some details from the treatment plans that were delivered. The four animals reported on in this paper were part of a larger animal study where several treatment parameters were varied. The effect of these parameters on treatment efficacy will appear in a subsequent publication.

**Table 1:** Treatment plan information.

				*MOSFET Pig*	
	*TLD Dog*	*MOSFET Dog #1*	*MOSFET Dog #2*	*Left Target*	*Right Target*
Plan Type	Conformal	Conformal	Isocentric	Isocentric	Isocentric
Prescription dose (Gy)	20	35	35	25	25
Collimator size (mm)	15	15	20	15	20
Nodes	69	39	29	23	23
Beams	150	89	29	23	23
MU	25699	22393	7737	6597	6386

**Figure 5:**
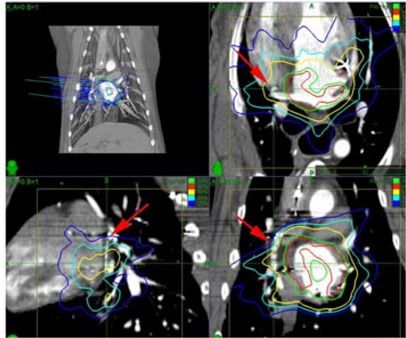
CT scan showing the TLD capsule sutured to the heart (red arrows). The capsule contained seven square TLD crystals bounded by two Teflon plugs. The capsule was positioned parallel to the spine on the right, posterior wall of the heart. The isodose contours show 21.4 Gy (red), 20 Gy (green), 12.6 Gy (yellow), 9.0 Gy (cyan), and 6.0 Gy (blue).

**Figure 6:**
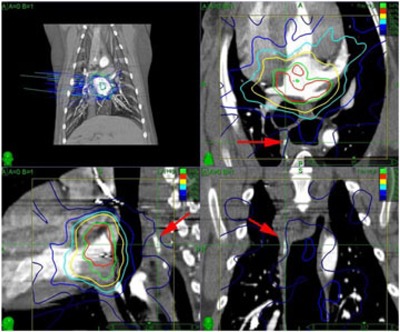
CT scan showing the TLD capsule sutured to the esophagus (red arrows). The capsule contained seven square TLD crystals bounded by two Teflon plugs. The capsule was positioned parallel to the spine on the left wall of the esophagus. The isodose contours show 21.4 Gy (red), 20 Gy (green), 12.6 Gy (yellow), 9.0 Gy (cyan), 6.0 Gy (blue), and 2.4 Gy (dark blue).

Each TLD was contoured in MultiPlan and the median dose off all points within the contour was recorded (see Figs. 5 and 6). The predicted doses for the heart TLDs ranged from 9 to 15 Gy, while the doses predicted for the esophagus TLDs ranged from 1 to 2 Gy. The treatment plan was delivered 56 days after the initial surgery. Five months after the radiation treatment, the dog was sacrificed and the TLDs were harvested. A relatively lengthy survival post‐treatment was required to allow for any lesions produced by the radiation to develop. Examination of the TLD capsules showed that bodily fluid had leaked onto the TLDs. The TLDs were rinsed in distilled water and air‐dried. The chips were then read by a commercial TLD reading service (Landauer Inc., Glenwood, IL).

Since the MOSFET sensors were not present in CT scans, their location was not known at the time of treatment planning. However, during CyberKnife treatment, the CyberKnife's stereotactic X‐ray system provided the location of the MOSFET sensors relative to the sutured fiducials. Since the sutured fiducials were visible in CT also, we determined the location of the MOSFET sensors in CT as follows.

**Figure 7:**
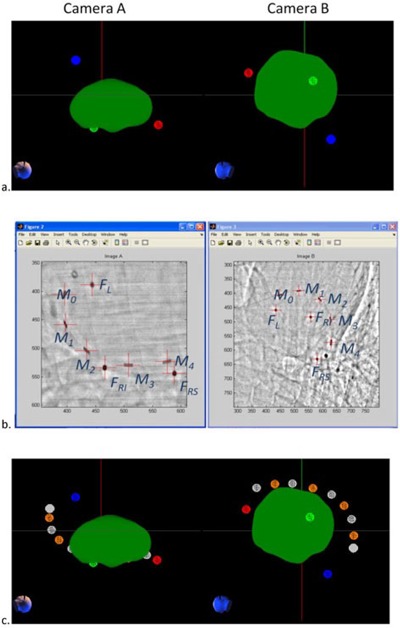
The method used to determine the dosimeter locations: (a) the locations of the sutured fiducials (red, blue, and green balls) in the CT image are projected to match the images from the CyberKnife system and show their spatial relationship to the prescription dose cloud (green); (b) enhanced CyberKnife X‐ray images showing the sutured fiducials ($F_{L}$, $F_{\text{RI}}$, $F_{\text{RS}}$) and the fiducials attached to the MOSFET flex ($M_{0}-M_{4}$); (c) positions of the MOSFET sensors (orange balls) relative to the fiducials on the MOSFET flex circuit (gray balls), the fiducials sutured to the epicardium (red, green, and blue balls), and the prescription dose cloud (green).

After the placement of the MOSFET dosimeters in the coronary sinus and the alignment of the animal in the CyberKnife system, but before the treatment had begun, the exact positions of the MOSFET sensors were determined using the CyberKnife's stereotactic X‐ray system. As shown in Fig. 7, the fiducials attached to the MOSFET array and the fiducials sutured to the epicardium were identified in both images and the CyberKnife imaging coordinates were determined. A rigid‐body transform was created to map the CyberKnife coordinates of the sutured fiducial positions to their CT coordinates. This same rigid‐body transform was then used to determine the locations of fiducials attached to the MOSFET array in CT space. The MOSFET dosimeters were located midway between the fiducials inside the flexible sheath. The MOSFET detector locations were estimated in the CT coordinate system using a spline interpolation. A spline was used as a convenient way to account for the gradual curvature of the array.

**Figure 8:**
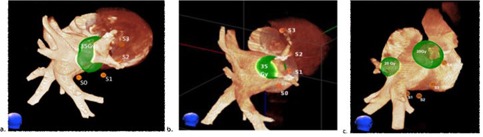
Reconstruction of the MOSFET sensor positions relative to the cardiac anatomy (from CT) for (a) dog 1, (b) dog 2, and (c) the pig. A single 35 Gy dose cloud was delivered to the main right pulmonary vein in each of the dogs, while two separate treatments were delivered to right and left pulmonary veins in the pig. The positions of the MOSFET sensors at the time of treatment are shown with orange balls labeled S0 (distal) to S3 or S4 (proximal). In the pig, a MOSFET sensor (S3) was much closer to the right target lesion than any of the sensors were to the left target lesion. In the pig (c), the distal sensor (S0) is hidden behind the pulmonary vein.

The position of the MOSFET array was then adjusted so that one of the MOSFET detectors was near the planned dose cloud. Additional X‐ray images were acquired during CyberKnife treatment. The location of the sensors was determined for each image pair and the average sensor location was used to predict the dose to the sensor. Figure 8 shows the locations determined for the MOSFET detectors in the two dogs and one pig studied.

### C. Calibration

The two TLD capsules that were not implanted in the dog were irradiated using a 6 MV linac X‐ray source (Trilogy, Varian Medical Systems Inc., Palo Alto, CA) one week after the animal was irradiated. One capsule, containing six crystals, was irradiated to 18 Gy and the other, containing seven crystals, was irradiated to 22 Gy. These capsules were retained at room temperature until the other capsules were harvested from the animal. In order to determine the effect of washing the *in vivo* crystals in distilled water, half of the calibration crystals were also washed in the same way. The calibration TLDs were processed at the same time as the *in vivo* TLDs. It was not feasible to maintain the calibration TLDs at body temperature for the five months between exposure and processing. The measurements could, therefore, have been affected by temperature dependence of dose fading. For LiF: Mg, Ti TLDs, Burgkhardt et al.^(^ ^15^ ^)^ measured dose fading of 5% over 50 days at 25°C and 15% over the same period at 50°C.

Because the first set of calibration TLDs received a much higher dose than the *in vivo* TLDs, a second set of calibration TLDs was irradiated at 2, 3, 9, 15, 18, and 22 Gy. Twelve TLDs were used to create six capsules in the same manner as before. Because of time constraints, these were read only five days after irradiation.

**Figure 9:**
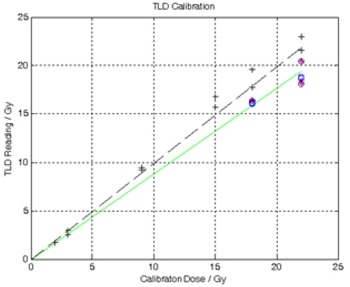
TLD Calibration. The first set of calibration TLDs were exposed at 18 and 22 Gy (blue circles and red asterisks). The TLDs in this first set that were washed (red asterisks) showed no significant difference from the unwashed TLDs (blue circles). The correction factor extrapolated from this calibration is shown as a green line. The second set of calibration TLDs were exposed at 2, 3, 9, 15, 18, and 22 Gy (black crosses). The correction factor calculated from this set of TLDs is shown as a black dashed line. This correction factor was then adjusted for loss of thermoluminescence over time to produce a fade‐corrected dose calibration.

Figure 9 shows the TLD calibration curve. The first set of TLDs showed that washing the TLDs with distilled water did not affect the measured dose. The calibration factor from these TLDs (subsequently called the extrapolated calibration) can be extrapolated to the range of the *in vivo* TLDs. The second calibration set (subsequently called the fade‐corrected calibration) provided data near the predicted doses of the *in vivo* TLDs, but were processed much sooner after the exposure (five days) than were the *in vivo* TLDs (five months).

The signal from TLDs is known to fade over time resulting in reduced readings when the time between exposure and reading is large.^(^ ^16^ ^)^ By calculating the ratio of the TLD readings for doses (18 and 22 Gy) that were calibrated with both five‐day and five‐month fade duration, the magnitude of the fading was estimated as 16%. A correction factor was calculated for the heart TLDs using the 9 and 15 Gy calibration doses adjusted for fading. A second correction factor for the esophagus TLDs was calculated based on the 2 and 3 Gy calibration doses adjusted for the fading. Any dose dependence of the TLD fading was neglected in this calibration.

The 13 TLDs used for the extrapolated calibration (those read after five months) can be used to estimate the precision of the TLD measurements. The standard deviation of the TLDs exposed to 18 Gy was 0.9% of their average value, while the standard deviation of the TLDs exposed to 22 Gy was 5.4% of their average value. As can be seen in Fig. 9, two of the six TLDs exposed to 22 Gy had significantly greater response. This variation may have appeared in the implanted TLDs, as well.

The MOSFET dosimeters were calibrated using the CyberKnife system in a configuration where one monitor unit delivered 0.01 Gy. The MOSFET array was sandwiched between solid water so that the photon beam passed through 1.5 cm of water equivalent before reaching the array and 4 cm after passing it. The X‐ray source was positioned 80 cm from the array with the beam normal to the surface of the solid water. Using a 6 cm collimator, 2.00 Gy was delivered to each element of the array four times. An average of the voltage change on the MOSFET was used as a calibration factor for determining the experimental dose. Because the MOSFET detectors were of the dual‐bias type, minimal temperature dependence was expected.^(^ ^17^ ^)^ The standard deviation of these calibration factors provided an indication of the inherent reproducibility of the MOSFET measurements. The standard deviations ranged from 1.2% to 1.8%, with an average standard deviation of 1.5%. This variation was expected on the *in vivo* dose measurements.

### D. Animal care

All animals were treated humanely and in accordance with the Animal Welfare Act (7 USC 2131) and the National Institutes of Health Guide for the Care and Use of Laboratory Animals. All activities involving animals were conducted with strict veterinary oversight and with full Institutional Animal Care and Use Committee approval at the Sutter Memorial Hospital Research Institute, Sacramento, CA. The research facility is certified by the United States Department of Agriculture (USDA), and is fully accredited by the Association for Assessment and Accreditation of Laboratory Animal Care International (AAALAC).

## III. RESULTS

All animals survived the procedures and demonstrated no adverse effects. Prior to sacrifice, ECG and transesophageal ultrasound evaluations were performed on each animal. These tests showed normal cardiac function.

The results of the TLDs placed on the epicardium are shown in Table 2. The TLDs were positioned at a small distance from the target area. Therefore, the predicted doses ranged from 46% to 73% of the prescription dose of 20 Gy. The measured doses were within 1 Gy of the predicted doses, except for the most caudal TLD where the measurement was 2.1 Gy lower than the predicted dose of 12.5 Gy. The relative difference between the measured and predicted doses ranged from 6% to −16.8%, with five of the seven measurements within 5% of the predicted value for either calibration measurement. The TLD measurements on the esophagus are shown in Table 3. The TLDs were positioned at some distance from the target, so doses and dose gradients were relatively small. The largest difference between measured and predicted was 1.2 Gy where the predicted dose was 2.9 Gy (using the extrapolated dose calibration with the most cranial TLD). The fade‐corrected calibration resulted in less deviation from the predictions than the extrapolated calibration. The TLD measurements were predominantly lower than the planned dose, but the differences were mostly within 1 Gy.

**Table 2:** Doses measured *in vivo* with TLDs in the canine heart.

		*Extrapolated Calibration*		*Fade‐Corrected Calibration*	
*Heart TLDs*	*Predicted Dose (Gy)*	*Measured (Gy)*	*Difference (%)*	*Measured (Gy)*	*Difference (%)*
Most caudal	12.5	10.8	−13.6	10.4	−16.8
	12.1	12.1	0.0	11.7	−3.3
	13.4	14.2	6.0	13.7	2.2
	14.1	13.9	−1.4	13.4	−5.0
	14.6	14.9	2.1	14.4	−1.4
	10.6	10.7	0.9	10.3	−2.8
Most cranial	9.2	8.9	−3.3	8.6	−6.5

**Table 3:** Doses measured *in vivo* with TLDs from the canine esophagus.

		*Extrapolated Calibration*		*Fade‐Corrected Calibration*	
*Esophagus TLDs*	*Predicted Dose (Gy)*	*Measured (Gy)*	*Difference (%)*	*Measured (Gy)*	*Difference (%)*
Most caudal	2.1	1.7	−19.0	2.0	−4.8
	2.2	1.6	−27.3	1.8	−18.2
	2.1	1.7	−19.0	2.0	−4.8
	2.0	1.5	−25.0	1.8	−10.0
	2.2	1.9	−13.6	2.2	0.0
	2.6	1.7	−34.6	2.0	−23.1
Most cranial	2.9	1.7	−41.4	2.0	−31.0

Tables 4 and 5 show comparisons between the MOSFET measurements and plan dose in the two dogs in which this dosimeter was used. Tables 6 and 7 show comparisons for the MOSFET measurements in the pig. Since the dosimeters were spaced apart by a large distance (approximately 2 cm), we were able to measure a large range of locations across the planned dose cloud. The highest doses measured were 13.9 Gy in the first dog, 22.0 Gy in the second dog, and 15.4 Gy in the pig. These corresponded to 40%, 63%, and 61% of the prescription doses of 35 Gy in the dogs and 25 Gy in the pig. The relative differences between measurements and predicted values were smaller for the higher doses (nearer the target). For points with doses above 9 Gy, the MOSFET measurements were within 7.5% of the predicted value.

**Table 4:** Measured vs. predicted doses for MOSFET measurement in dog 1. The treatment was isocentric with a prescription dose of 35 Gy to 83% of the maximum dose.

*Sensor ID*	*Predicted Dose (Gy)*	*Measured Dose (Gy)*	*Difference (%)*
S0	1.03	0.98	−4.9
S1	8.74	11.70	33.9
S2	14.59	13.90	−4.7
S3	14.21	13.60	−4.3
S4^a^		1.30	

a The exact location of S4 could not be determined in this experiment, so no dose could be predicted for it.

**Table 5:** Measured vs. predicted doses for MOSFET measurement in dog 2. The treatment was isocentric with a prescription dose of 35 Gy to 83% of the maximum dose.

*Sensor ID*	*Predicted Dose (Gy)*	*Measured Dose (Gy)*	*Difference (%)*
S0	9.69	9.50	−19.0
S1	23.79	22.00	−7.5
S2	1.03	1.07	3.9
S3	0.38	0.29	−23.7
S4^a^		0.14	

a The exact location of S4 could not be determined in this experiment so no dose could be predicted for it.

**Table 6:** MOSFET results from porcine experiment, right target lesion.

*Sensor ID*	*Predicted Dose (Gy)*	*Measured Dose (Gy)*	*Difference (%)*
S0	0.33	0.32	−3.0
S1	0.31	0.31	0.0
S2	0.35	0.40	14.3
S3	1.77	2.52	42.4
S4	16.32	15.40	−5.6

**Table 7:** MOSFET results from the porcine experiment, left target lesion.

*Sensor ID*	*Predicted Dose (Gy)*	*Measured Dose (Gy)*	*Difference (%)*
S0	0.66	1.46	121.2
S1	1.26	1.15	−8.7
S2	0.56	0.67	19.6
S3	3.26	1.81	−44.5
S4	0.23	0.30	30.4

## IV. DISCUSSION

This represents the first published report of *in vivo* dose measurement during cardiac ablation with radiosurgery.

The doses measured *in vivo* were very close to the planned dose. The heart doses measured with TLDs using the extrapolated calibration averaged 1% lower than the plan dose, while using the fade‐adjusted calibration the measurements were, on average, 5% lower. The extrapolated calibration was based on chips that were irradiated and processed with the same interval as the test chips but at higher doses, while the fade‐adjusted calibration was based on chips irradiated at similar doses but processed much sooner after irradiation than the test chips. The better agreement with the extrapolated calibration may indicate that the fade correction applied led to more error than extrapolating the dose from chips that were aged appropriately but calibrated at a higher dose. Despite these calibration issues, the agreement between measurement and prediction is quite close considering TLD variation of approximately 2%^(^ ^18^ ^)^ and reproducibility of the TLDs estimated using the calibration chips at between 1% and 5%.

The doses measured at the esophagus did not agree with the plan dose as well as in the heart. Using the extrapolated calibration, the measurements were on average 25% lower than predicted. With the fade‐adjusted calibration, the measurements were on average 14% lower. The extrapolated dose calibration was based on measurements at 18 and 22 Gy, so it could be expected to be less reliable for doses less than 3 Gy than for doses in the 9 to 15 Gy range.

There were sources of uncertainty in the TLD measurements. The contamination of the *in vivo* TLDs with bodily fluid could have caused an erroneous reading. Surface contamination could have absorbed light emitted by the TLD before it was detected in the reading process, reducing the measured dose. Alternatively, contaminants could have created additional light as they burned in the 300°C oven used to read the TLD. This would have resulted in an erroneously high dose measurement.

The MOSFET measurements agreed well with the planned dose. Except for sensor S1 in dog 1, the difference between predicted and measured doses was less than 10% in locations receiving more than 5 Gy. Consorti et al.^(^ ^19^ ^)^ considered the overall uncertainty in the response of similar MOSFET dosimeters to be 3.5% and measured differences from predicted values less than 5% in most cases in intra‐operative clinical treatments (the largest difference was 11.7%).

Much of the uncertainty in the MOSFET comparison came from the difficulty in identifying the position of the MOSFET in the dose cloud. Because the sensors themselves could not be seen in the X‐ray images, differences between the actual catheter shape and the cubic hermite spline that was used to estimate the MOSFET position would result in a dose error due to the high gradients involved. Furthermore, the mapping of MOSFET positions from the CyberKnife X‐rays to CT assumed a rigid relationship between the sutured fiducials and the MOSFET sensors. The reproducibility, as estimated during MOSFET calibration, would be expected to contribute to between 1% and 2% of the variation seen.

There are also possible differences between the predicted and delivered doses. Errors in the respiratory tracking or the untracked cardiac motion could have affected dose delivery. Errors in beam targeting or the positioning of the animal could have resulted in dose delivery inaccuracies. Errors in the dose model used to accommodate the contrast agent could also have resulted in a difference between the predicted and delivered doses. The planning system used a ray‐tracing–based method to calculate the dose. This method has been shown to overestimate dose in the thorasic region due to dose rebuildup. The dose delivered to the esophagus could have been affected by spatial averaging caused by relative motion between the heart and esophagus. However, the dose gradient (as shown in Fig. 6) was very small in this region, which would minimize this effect.

## V. CONCLUSIONS

Both dosimeter methodologies tested in these animal models of cardiac ablation documented that doses delivered corresponded to predicted dose‐to‐volume plans for the cardiac targets to within about 10%. This is greater than the generally accepted +5% target for therapeutic dose delivery accuracy, but dose measurement uncertainty could easily account for the additional deviation. This showed that target motion, with its respiratory and cardiac components, did not strongly affect the dose delivery accuracy.

Although uncertainty in dose measurement and dose prediction could have partially offset one another, the agreement of these methods was strongly suggestive that the actual dose delivered was near the predicted dose. The general agreement between the *in vivo* measurements and the planned dose suggests that treatment planning and CyberKnife dose delivery for pulmonary vein ablation in the heart are accurate to within 10%.

## ACKNOWLEDGMENTS

CyberHeart would like to thank Dr. Vivek Reddy for his help with the pulmonary vein motion measurement (Fig. 1).
